# Direct cell-to-cell spread of a pathogenic yeast

**DOI:** 10.1186/1471-2172-8-15

**Published:** 2007-08-16

**Authors:** Hansong Ma, Joanne E Croudace, David A Lammas, Robin C May

**Affiliations:** 1 Molecular Pathobiology, School of Biosciences, University of Birmingham, Edgbaston, Birmingham, B15 2TT, UK; 2 Division of Immunity and Infection, The Medical School, University of Birmingham, Edgbaston, Birmingham, B15 2TT, UK

## Abstract

**Background:**

Cryptococcosis, a fatal fungal infection of the central nervous system, is one of the major killers of AIDS patients and other immunocompromised hosts. The causative agent, *Cryptococcus neoformans*, has a remarkable ability to 'hide' and proliferate within phagocytic cells of the human immune system. This intracellular phase is thought to underlie the ability of the pathogen to remain latent for long periods of time within infected individuals.

**Results:**

We now report that *Cryptococcus *is able to undergo 'lateral transfer' between phagocytes, moving directly from infected to uninfected macrophages. This novel process was observed in both *C. neoformans *serotypes (A and D) and occurs in both immortalised cell lines and in primary human macrophages. Lateral transfer is independent of the initial route of uptake, since both serum-opsonised and antibody-opsonised *C. neoformans *are able to undergo direct cell-to-cell transfer.

**Conclusion:**

We provide the first evidence for lateral transfer of a human fungal pathogen. This rare event may occur repeatedly during latent cryptococcal infections, thereby allowing the pathogen to remain concealed from the immune system and protecting it from exposure to antifungal agents.

## Background

*Cryptococcus neoformans *is an encapsulated basidiomycete yeast that causes disseminating infections in immunocompromised hosts, especially those with AIDS. There are two varieties of *C. neoformans*: *C. neoformans *var. *neoformans *(Serotype D) and *C. neoformans *var. *grubii *(Serotype A). Both are ubiquitous in the environment and can be commonly isolated from avian excreta, soil and trees. Infection is thought to begin with the inhalation of airborne spores and epidemiological evidence suggests that exposure to *Cryptococcus *early in life can produce a prolonged, asymptomatic, latent infection [1]. Should an infected individual later become immunocompromised, the fungus can then spread from the lungs to the central nervous system to cause meningoencephalitis, which is uniformly fatal without rapid clinical intervention [2].

The mechanism by which *C. neoformans *achieves latency and persistence prior to dissemination from its primary site of infection in the lung remains poorly understood. Recent data have revealed an important role for macrophages in this process. Firstly, *C. neoformans *shows a remarkable ability to survive and proliferate within host macrophages *in vitro *[3,4]. Secondly, live cryptococci can be recovered from circulating monocytes in infected mice [5]. Thirdly, recent studies by our group and others have demonstrated that *C. neoformans *is able to escape from within macrophages by a novel expulsive mechanism [6,7]. After the expulsion, both the host macrophage and the expelled *C. neoformans *appear morphologically normal and continue to proliferate, suggesting that this process may represent an important mechanism by which pathogens are able to escape from phagocytic cells without triggering host cell death and thus inflammation.

These findings have led to the so-called "Trojan horse" hypothesis on dissemination of *C. neoformans*, which proposes that cryptococci are engulfed by phagocytic cells at an early stage of infection and then trafficked by these host cells into distal tissues without being exposed to the full onslaught of the immune system [8,9]. However, it is not known how cryptococci remain intracellular for prolonged periods prior to dissemination, given that the period of latency far exceeds the natural lifespan of a host macrophage.

Using timelapse microscopy, we now show that *C. neoformans *is able to undergo 'lateral transfer' between phagocytes, during which the pathogen moves directly from an infected cell to neighbouring uninfected cells. This mechanism may explain the ability of *C. neoformans *to remain latent within the host during long periods of asymptomatic persistence, as well as providing protection during dissemination from primary sites of infection.

## Results

**Figure 1:**
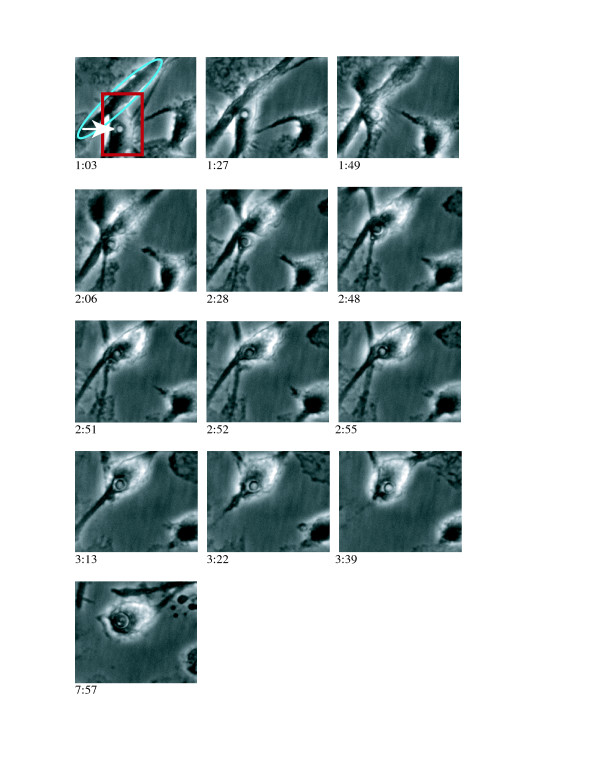
**Lateral transfer of JEC21 (white arrow) from infected (donor, rectangle) to uninfected (recipient, oval) human primary cells**. (A to E) The donor cell moves underneath the recipient cell and after 85 min, the recipient macrophage contacts the cryptococcal containing compartment. (F to I) About 160 min after the onset of filming, membrane fusion starts to occur at the contact point of the two cells and initiates lateral transfer of the yeast from the donor cell to the recipient cell. The whole process takes only seven minutes. (J to M) Upon completion, the cryptococcal cell is entirely in the recipient macrophage and the donor macrophage moves away.

We observed lateral transfer in both cultured murine J774 cells and human primary macrophage cells [see additional files 1 and 2]. During this process *C. neoformans *cells contained within a membrane-bound compartment of an infected macrophage are passed directly to a neighbouring (uninfected) macrophage (Figure 1). Compared to expulsion [6,7] and intracellular proliferation [4], lateral transfer is a rare event since we observed only four events of lateral transfer after monitoring 177 human primary macrophages with internalised cryptococci (Table 1). However, we are likely to underestimate the true rate of lateral transfer, since experimental constraints mean that we can only monitor infected cells for sixteen hours after phagocytosis.

**Table 1:** The rate of expulsion and lateral transfer events recorded for JEC21 (Serotype D) in primary human macrophage cells

	Primary human macrophage cells
Total number of macrophages observed	661
Macrophages with internalized yeast	177 (26.8%)
Occurrence of cryptococcal expulsion	47 (26.6%)
Occurrence of lateral transfer	4 (2.3%)

Like cryptococcal expulsion [6,7], lateral transfer is independent of the initial route of uptake, since both serum-opsonised and antibody-opsonised *C. neoformans *are able to undergo direct cell-to-cell transfer (data not shown). We have also observed lateral transfer in two *C. neoformans *strains: JEC21 (Serotype D) and 125.91 (Serotype A), suggesting that it is not serotype dependent.

Lateral transfer is preceded by highly dynamic movement of both the donor and recipient macrophage, which is driven by the actin cytoskeleton. Imaging of over 300 macrophages treated with the actin depolymerising drug cytochalasin D revealed no cases of lateral transfer, suggesting that transfer is dependent either on actin dynamics *per se*, or on actin-dependent host cell motility.

Lateral transfer is a very rapid process; transfer always completes within ten minutes of the onset of cell membrane fusion between the donor and recipient macrophage cells. Interestingly, three out of four lateral transfer events observed in primary human cells were followed by yeast expulsion, suggesting that the cryptococcal phagosome may be in a special, highly fusogenic state. Alternatively, it is possible that cryptococci that subsequently undergo lateral transfer are initially internalized into an atypical 'transfer-competent' compartment (rather than a phagosome) although this seems unlikely given that internalized cryptococci have previously been shown to acquire normal phagosome markers [7,10].

## Discussion

In this study, we report a direct cell-to-cell spread mechanism used by *C. neoformans *to travel between macrophage cells. The molecular mechanism that drives lateral transfer needs further investigation. It superficially resembles the phenomenon of cryptococcal expulsion, since both phenomena appear to be driven by rapid membrane fusion. However, since lateral transfer is blocked by cytochalasin D and cryptococcal expulsion is not [6,7], it appears that there may be important mechanistic differences between the two processes which may reflect a greater dependence on host cell motility in the case of lateral transfer. Direct cell-to-cell spread has been described for some pathogenic bacteria, such as *Listeria, Rickettsia *and *Shigella*, and many viruses (e.g. poxvirus). Most of these organisms undergo direct cell-to-cell spread via polarised actin polymerisation, which generates force to propel them through the cytoplasm of the infected cell and into uninfected neighbouring cells [11-13]. However, the transmission of cryptococci between cells is morphologically very different to these other pathogens and is not preceded by directed propulsion within the host cytoplasm, suggesting that the underlying mechanism may be distinct.

Despite the low rate of lateral transfer observed *in vitro*, we hypothesise that this process may have significant clinical implications since it allows *C. neoformans *to remain intracellular, thus avoiding immune recognition. Furthermore, it allows the pathogen to move from weak to healthy phagocytes, thus ensuring intracellular persistence of the pathogen even if the host cell starts to die. Finally, infected macrophage cells may travel widely throughout the host circulatory and lymphatic systems, where they interact intimately with one another and with other cell types through transient contacts [12]. We speculate that internalised *C. neoformans *may use such transient contact in order to cross the blood-brain barrier by direct cell-to-cell spread from adherent infected macrophages to microvascular endothelial cells [14]. In fact, spreading from macrophages to other cell types during dissemination has been demonstrated for other pathogens *in vitro*. For instance, the Gram-positive bacterium *Listeria monocytogenes *can infect neurons by cell-to-cell spread from adherent macrophages, a more efficient process than direct invasion of neurons [15]. Intriguingly, cell-to-cell spread of bacteria from adherent infected phagocytes to endothelial cells of the central nervous system has also been reported [8,16] and it will clearly be of great interest to investigate whether a similar process may occur during cryptococcosis.

## Conclusion

We report a new phenomenon, termed lateral transfer, by which pathogenic yeast can be transmitted between host cells. Lateral transfer of *Cryptococcus *is likely to be an important step regulating phagocyte-facilitated latency and dissemination. A better understanding of this process will be of considerable importance in developing new therapeutic strategies against cryptococcosis.

## Methods

### Yeast Strains and Growth Conditions

*Cryptococcus neoformans *strains JEC21 (Serotype D) and 125.91 (Serotype A, Tanzanian clinical isolate) were grown overnight in YPG medium (1% yeast extract, 1% peptone, and 2% glucose) with 50 μg/ml ampicillin at 25°C with shaking.

### Cell Line and Culture Media

J774 cells were grown at 37°C in 5% CO_2 _in DMEM with 10% heat-inactivated FBS, 2 mM L-glutamine, 100 units/ml penicillin, and 100 μg/ml streptomycin. The cell line was used between five and 20 passages after thawing.

Human primary blood macrophage cells (PBMC) were prepared as described previously [17] and resuspended at 6 × 10^6 ^cells/ml in RPMI 1640 medium containing 2% pooled AB^+ ^male human serum and 2 mM L-glutamine. The PBMC were then seeded into 175 cm tissue-culture flasks. Non-adherent cells were removed by extensive washes with PBS, and adherent monocytes were incubated overnight in RPMI medium + 5% human serum supplemented with GM-CSF (100 IU/ml). After overnight incubation, adherent cells were removed from flasks by incubation on ice in pre-chilled PBS and cultured for 4–5 days in RPMI + 5% human serum and GM-CSF (100 IU/ml). The cells were adjusted to 1 × 10^6 ^cells/ml and then aliquoted into 3 cm plastic Petri dishes in 3 ml cultures. The cell media was replenished on days 3 and 5 and yielded adherent, confluent macrophage cultures at day 7. Subsequently, the macrophages were activated with LPS (1 μg/ml) and IFNγ (1000 IU/ml), which were added to the culture dishes 24 hr prior to infection with *C. neoformans*.

### Phagocytosis Assay

J774 cells (6 × 10^5^) were plated into a 35 mm tissue-culture-treated plate 16–24 hr before the assay. Shortly before use, cells were incubated for 1 hr in serum-free DMEM medium (Complete DMEM medium without heat-inactivated FBS) containing 150 ng/ml PMA. Similarly, the primary macrophage cells (which have been activated with LPS and IFNγ) were incubated for 1 hr in serum-free DMEM medium.

At the same time, *C. neoformans *cells were washed three times with phosphate-buffered saline (PBS) [pH 7.2] and counted in a haemocytometer. For experiments using J774, *C. neoformans *was incubated with 10 μg/ml of the monoclonal antibody 18B7 (a kind gift of Arturo Casadevall) at 37°C for 1 hr. 20% fresh human serum was used to opsonise *C. neoformans *for experiments with human primary macrophages.

To commence the assay, the medium containing PMA was removed and replaced by normal serum-free medium containing *C. neoformans *at a ratio of ten yeast cells per macrophage. We allowed phagocytosis to proceed for 2 hr at 37°C in a 5% CO_2 _atmosphere. We then removed non-internalized yeast cells by three successive washes with serum-free medium and maintained cells in serum-free medium with 25 mM HEPES for time-lapse imaging.

### Image Capture and Analysis

Cells were maintained at 37°C with a temperature-controlled chamber (Solent Scientific) and imaged on a Zeiss Axiovert 100 inverted microscope with a 32× dry objective lens. Time-lapse movies were made with OpenLab (Improvision), capturing one frame every 90 s for 16 h on a QICAM camera. The number of lateral transfer event was counted by eye.

For producing Figure 1 and Additional Files 1 and 2, the original time-lapse movie was decompiled into individual TIFF images with ImageJ. These were then cropped to the region of interest and sharpened. A semiopaque mask and arrow were added to the first frame (to indicate the cell of interest) with Adobe Photoshop 7.0 before the TIFF image stack was recompiled into a QuickTime movie with ImageJ.

## Note added in proof

The publication of this article was coordinated with the publication of 'Cell-to-cell spread and massive vacuole formation after Cryptococcus neoformans and infection of murine macrophages' by Alvarez and Casadevall in *BMC Immunology *[18].

## Authors' contributions

HM carried out all the experiments. JEC and DAL prepared primary macrophage cultures. Experimental design and manuscript preparation were carried out by HM and RCM. All authors have read and approved the manuscript.

## Supplementary Material

Additional file 1 Lateral transfer of JEC21 from an infected to an uninfected human primary macrophage. The infected (donor) cell is highlighted in the first frame. The donor cell moves underneath the recipient cell and, at 1:25, the recipient macrophage contacts the cryptococcal phagosome. About 160 min after the onset of filming, membrane fusion starts to occur at the contact point of the two cells and initiates lateral transfer of the yeast from the donor cell to the recipient cell. The whole process takes only seven minutes. Upon completion, the cryptococcal cell is entirely in the recipient macrophage and the donor macrophage moves away. Click here for file

Additional file 2 Lateral transfer of *Cryptococcus neoformans *strain 125.91 between J774 murine-derived macrophages. The donor cell (highlighted in frame one, upper right) transfers one of the two internalized cryptococci to the recipient cell (bottom left). The transferred *Cryptococcus *is marked by a white arrow in frame one. The donor cell moves downwards, contacting the recipient macrophage approximately 90 minutes after the onset of filming. Transfer occurs approximately 115 minutes into the movie. Note that the transferred *Cryptococcus *then moves underneath a surface-bound cryptococcal cell (clearly visible at 124 minutes), demonstrating that it is held within an intracellular compartment and not simply attached to the plasma membrane. Click here for file
